# Supplementary material for: Prognostic Value of Chemotherapy Response Score (CRS) Assessed on the Adnexa in Ovarian High-Grade Serous Carcinoma: A Systematic Review and Meta-Analysis
Source: Diagnostics (Basel). 2022 Mar 4;12(3):633. doi: 10.3390/diagnostics12030633 (PMC8946962; doi:10.3390/diagnostics12030633)
Supplement: Supplementary file 1 [file diagnostics-12-00633-s001.zip › diagnostics-1616781-supplementary.pdf]

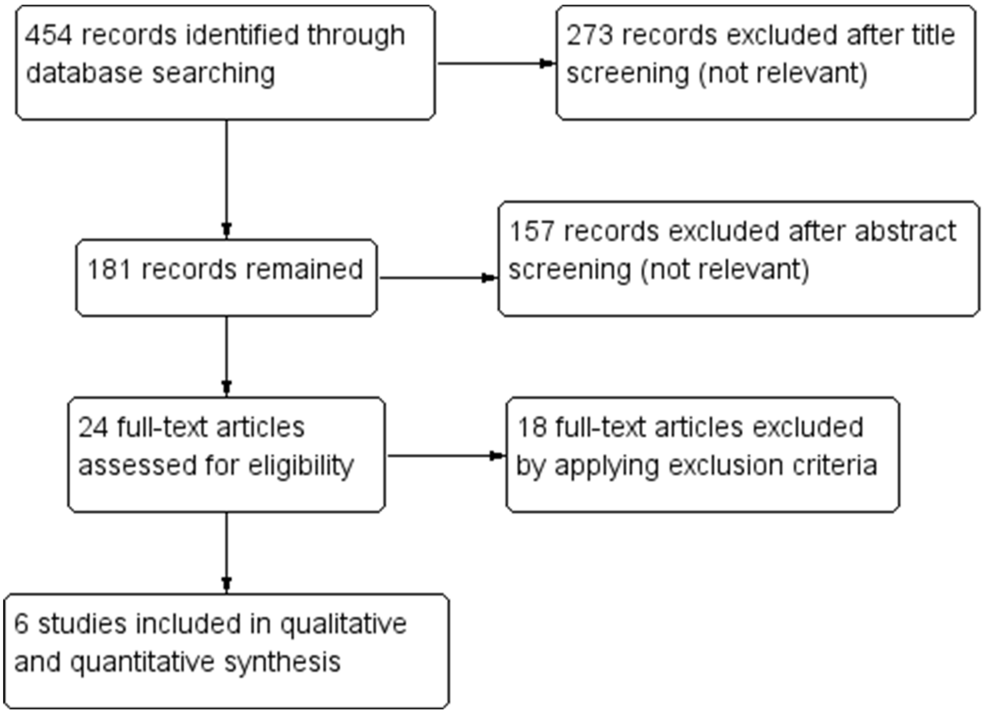

Figure S1. Flow diagram of studies identified in the systematic review (Preferred Reporting Item for Systematic Reviews and Meta-analyses (PRISMA) template).
